# Can Human Movements Explain Heterogeneous Propagation of Dengue Fever in Cambodia?

**DOI:** 10.1371/journal.pntd.0001957

**Published:** 2012-12-06

**Authors:** Magali Teurlai, Rekol Huy, Bernard Cazelles, Raphaël Duboz, Christophe Baehr, Sirenda Vong

**Affiliations:** 1 Epidemiology and Public Health Unit, Institut Pasteur du Cambodge, Phnom Penh, Cambodia; 2 IRD UMR LOCEAN, UMR ESPACE-DEV, New-Caledonia, France; 3 National Dengue Control Program, National Centre for Parasitology, Entomology and Malaria Control, Ministry of Health, Phnom Penh, Cambodia; 4 Ecologie & Evolution, UMR 7625, CNRS-UPMC-ENS, Paris, France; 5 UMMISCO UMI 209 IRD - UPMC, Bondy, France; 6 CIRAD UPR Agirs, Montpellier, France; 7 Météo France, CNRM, Toulouse, France; 8 CNRS, GAME URA 1357, Toulouse, France; Duke University-National University of Singapore, Singapore

## Abstract

**Background:**

Determining the factors underlying the long-range spatial spread of infectious diseases is a key issue regarding their control. Dengue is the most important arboviral disease worldwide and a major public health problem in tropical areas. However the determinants shaping its dynamics at a national scale remain poorly understood. Here we describe the spatial-temporal pattern of propagation of annual epidemics in Cambodia and discuss the role that human movements play in the observed pattern.

**Methods and Findings:**

We used wavelet phase analysis to analyse time-series data of 105,598 hospitalized cases reported between 2002 and 2008 in the 135 (/180) most populous districts in Cambodia. We reveal spatial heterogeneity in the propagation of the annual epidemic. Each year, epidemics are highly synchronous over a large geographic area along the busiest national road of the country whereas travelling waves emanate from a few rural areas and move slowly along the Mekong River at a speed of ∼11 km per week (95% confidence interval 3–18 km per week) towards the capital, Phnom Penh.

**Conclusions:**

We suggest human movements – using roads as a surrogate – play a major role in the spread of dengue fever at a national scale. These findings constitute a new starting point in the understanding of the processes driving dengue spread.

## Author Summary

Dengue fever is a mosquito borne viral infection. It has become a major public health problem during the past decades: only 9 countries were affected in the 1970s; dengue is now endemic in more than 100 countries. In the absence of any vaccine or specific treatment, control of dengue fever is currently limited to vector control measures, which are difficult to implement and hardly sustainable, especially in low income countries. To implement efficient control measures, it is crucial to understand the dynamics of propagation of the disease and the key factors underlying these dynamics. In this study, data from 8-year national surveillance in Cambodia were analysed. Dengue fever follows a recurrent pattern of propagation at the national scale. The annual epidemics originate from a few rural areas identified in this work. This study also suggests additional evidence for the role of human movement in the spatial dynamics of the disease, which should be accounted for in control measures. These results differ from the current knowledge about dengue dynamics and are therefore of interest for future research.

## Introduction

Cambodia is a low-income tropical country, hyper-endemic for all four serotypes of dengue infection. As such, dengue epidemics occur every year during the rainy season and result in considerable morbidity and economic burden. The basis for these recurrent epidemics is an increased vector activity during the rainy season and complex interactions between hosts and viruses with short lived cross-protective immunity [1]–[3]. In the absence of a vaccine, control is limited to vector control measures. Regarding dengue dynamics, locally, dengue outbreaks are explosive and tend to be focal, perhaps reflecting the limited dispersal of the vector, which visits few houses in a life-time, and have a limited flight range [4], [5]. At an international scale, human movement is known to be a major factor responsible for the virus transportation among big urban centres [6]. At a national scale, little is known about the spatial propagation of the disease. In many endemic countries, urban centres are thought to act as a reservoir of the virus from where it can spread to the rest of the country [6]–[9]. In Thailand [7] and more recently in Southern Vietnam [8], researchers demonstrated the existence of a travelling wave either within the 3-year periodic mode or the annual mode of oscillation. However, the underlying factors responsible for these waves remain unknown [1], [7]–[10]. Hypotheses include immunological host-virus interactions, differences in virus virulence, or heterogeneity of the spatial distribution of the host population [1]. Synchronisation of cases has also been observed in the 3 year periodic band in Thailand [7]. Mechanisms responsible for higher synchronicity could include climatic forcing [8], [10].

The objective of this study was to determine whether there was a spatial-temporal pattern of dengue propagation in Cambodia repeating year after year. The characterisation of such patterns is important to understand the forces driving dengue spatial spread and aid better control and logical allocation of public health resources.

## Methods

### Study area

**Figure 1:**
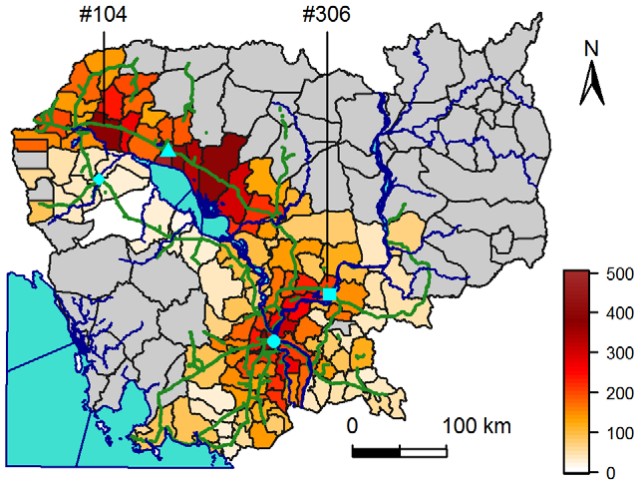
Map of mean annual dengue fever incidence rates in districts of Cambodia. Mean annual incidence rates (in number of cases declared per 100,000 people per year) are calculated over 2002 to 2008, for districts with more than 20 people per km^2^. Cambodia is surrounded by the Indian Ocean (bottom left), Thailand (West), Lao (North) and Vietnam (East and South-East). Phnom Penh, the capital, is represented by a circle, Siem Reap by a triangle, Kampong Cham by a square and Battambang by a lozenge. Blue lines represent the Mekong River, going north to south, and the Tonle Sap River linking the Tonle Sap central Lake to the Mekong River. Green lines represent national roads. Grey districts have less than 20 people per km^2^.

Cambodia is a low-income country located in South-East Asia, divided into 24 provinces and 180 districts covering 181,035 km^2^. Out of the 13.4 million people in 2008, more than 80% live in rural areas; 1.3 million people (9.9%) live in Phnom Penh, the capital city. Cambodia is lagging behind other countries in South-East Asia in terms of economic or demographic development. For example, unlike Thailand, the demographic transition has not occurred yet. The South-West and the North of the country (see grey districts in Figure 1) are mountainous regions with low population density (mean district density of 18 people per km^2^). The rest of the country is composed of flat plains with few cities (mainly Phnom Penh, Kampong Cham, Siem Reap and Battambang, see Figure 1) scattered among rural areas. The weather is warm all year long, and the climate is dominated by the annual monsoon cycle, with a dry season (December to April) alternating with a wet season (May to November). Climatic variation from one area to another is limited. Temperature is homogeneous across the country and ranges annually from 21°C to 35°C. As a result, mosquitoes can be active all year long when considering only temperature. Rainfall or water availability are more likely to be the factors limiting the vector's activity.

### The data

Cambodian National surveillance recorded 109,332 dengue cases during 2002–2008, a period over which the reporting process was stable. Cases were reported passively from public hospitals and actively from 5 major sentinel hospitals located in the cities of Siem Reap (1 hospital), Kampong Cham (1 hospital) and Phnom Penh (3 hospitals). Cases were clinically diagnosed using the 1997 World Health Organization (WHO) case definitions, allowing clinical and paraclinical (haematocrit and platelets count) distinction between classic dengue fever, dengue haemorrhagic fever and dengue shock syndrome [2]. Of note, the new WHO case definition was only introduced in 2009 [11]. To increase specificity, only severe cases (e.g. dengue haemorrhagic fever) affecting children less than 16 years old were recorded in the database. Assuming that epidemic patterns of dengue would be stochastic in low population density areas, we excluded the 45 (/180) districts with less than 20 people per km^2^ from the analysis, dismissing 3298 declared cases (3.03% of the total declared cases). Since patients' districts of residence were recorded, we calculated weekly incidence rates for each of the 135 remaining districts. Denominators for incidence rates were interpolated linearly using the 1998 and 2008 national censuses. Based on age distribution similarities between provinces, and on similarities between 1998 and 2008 age structures, we assumed that age structure was homogeneous over the country and have not standardised incidence rates according to age. As national surveillance data were made available to be utilised for such temporal and spatial analyses and have been routinely published nationally and once internationally [2] no specific approval was requested from the Ministry of Health's National Ethics Committee. Moreover, data that were provided by the National Programme were anonymised prior to transfer to the Institut Pasteur du Cambodge. We subsequently randomly assigned new codes to each record and deleted the previous ones to unlink the present dataset to the national database.

### Temporal analysis

**Figure 2:**
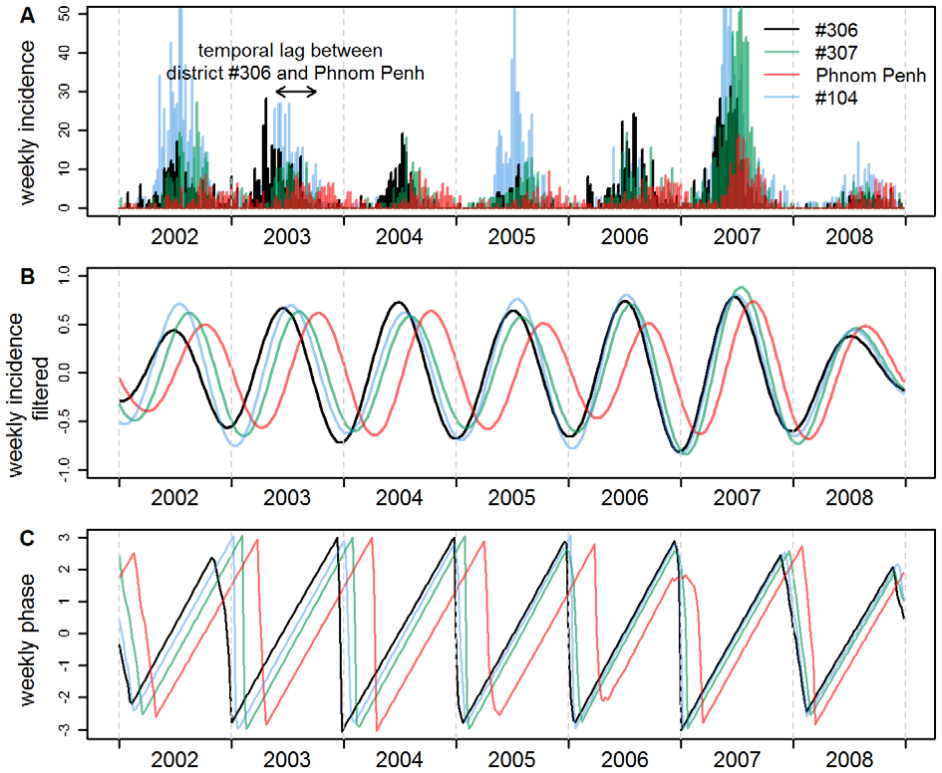
Weekly raw incidence rates, filtered incidence and phase of four districts in Cambodia. The four districts are: district #306 (black), a rural district located around Kampong Cham; Phnom Penh (red); District #307 (green), a rural district located mid-way between #306 and Phnom Penh; District #104 (blue). (A) Weekly raw incidence rates (in number of cases declared per 100,000 people per week). (B) Annual component of incidence, obtained by filtering raw weekly incidence in the 0.8–1.2 year periodic band using wavelet analysis. (C) Phase of the annual component of incidence, computed in the 0.8–1.2 periodic band using wavelet analysis (see Methods).

Time series of dengue incidence were square-root transformed to stabilise the variance, subsequently centred to zero-mean and normalised to unit variance. We then performed a wavelet transform and used wavelet phase analysis to describe dynamic patterns of dengue. This spectral method is well-suited for the analysis of non-stationary time-series such as epidemic curves. It is particularly useful to filter the data in any given frequency band and extract the phase of any given periodic component [10], [12]–[14] (see Figure 2 for an illustration of the method).

The wavelet transform was done using R software [15] and functions translated from Cazelles' Matlab toolbox. We used a Morlet wavelet as the mother wavelet. All equations used and vocabulary relative to wavelet analysis are detailed in [10], [12]–[14].

The wavelet coefficients corresponding to a period ranging from 0.8 to 1.2 year were used to reconstruct filtered time series corresponding to the annual epidemic in each district. These filtered time series, called “annual component of incidence”, are illustrated in Figure 2B.

The phases of the epidemic have been computed in the periodic band 0.8–1.2 year (see equation (5) in [10]), thus obtaining, for each district, a single time series of the phase of the cyclic annual component of the epidemic (Figure 2C). This phase can be viewed as the timing of the epidemics, and is almost not influenced by the intrinsic value of incidence. For a given week, if the seasonal epidemic occurs at the same time in two districts, the two annual components have the same phase. Another example in Figure 2, district #306 (a rural district located around Kampong Cham) has an advanced phase compared to Phnom Penh: the epidemic in Phnom Penh is lagging behind the one in district #306.

By calculating phase differences, one can then determine in which order districts are affected by the annual epidemic. This ranking allowed us to identify districts hit early by the seasonal epidemic. Time series of the temporal lag between seasonal epidemics at different locations were thus estimated from phase differences, according to equations (6)–(8) in [10]. This temporal lag is proportional to the phase difference.

In some districts identified as early districts, there were not enough cases reported to be confident that there was significant dengue transmission ongoing, given that cases are declared on a clinical basis. Therefore, for each district, we identified epidemic years using the national epidemic threshold [2], [16]. The national weekly threshold [2] for an epidemic was calculated as two standard deviations above a three weeks sliding mean computed over the weekly national incidence rates of the five past available non epidemic years [16] (from 2002 to 2006 in our study). We excluded from the analysis time periods from districts with low incidence, only including epidemic years defined as districts with a weekly incidence rate from January to December above the national threshold for an epidemic during two consecutive weeks. Subsequent analyses were restricted to the years with detected epidemics.

### Spatial analysis of dengue propagation

For a given year, to evaluate the speed at which dengue epidemic propagates along a given geographical axis comprised of I districts, we performed a regression analysis: Y_i_ = β0+β1 X1_i_+ε_i_ , with i in [1, I], Y_i_ the annual mean of the temporal lag between district i and a district located along the axis and selected as a time reference to compute temporal lags, and X1_i_ the corresponding distance as the crow flies, in km, between the centres of district i and the reference district. The speed of dengue propagation along the axis was estimated as the inverse of the regression slope β1.

To compare the dynamic pattern along J different axes, we performed, each year, an analysis of covariance [17]: Y_ij_ = β0_j_+β1_j_ X1_ij_+ε_ij_, with i in [1, I_j_], j in [1, J], Y_ij_ the annual mean of the temporal lag between district i of axis j and a district located along the axis j and chosen as a time reference, X1_ij_ the corresponding distance separating the centres of districts i and the reference district on axis j, and β0_j_ and β1_j_ the intercept and slope of the regression line of axis j. We will call “p-value of interaction” the p-value of the hypothesis that the model slopes β1_j_ are all equal, or in other words, that the speed of propagation is the same along different axes. If the model gave a p-value of interaction below 0.05, the propagation was considered heterogeneous along the different axes, and separate regressions were performed for each axis.

As we wanted to know whether the results were consistent from year to year, we performed this analysis each year.

All analyses and figures were performed using R software [15].

All confidence intervals (C.I.) provided were calculated using classical methods for calculating a confidence interval around a mean, unless stated otherwise.

## Results

**Figure 3:**
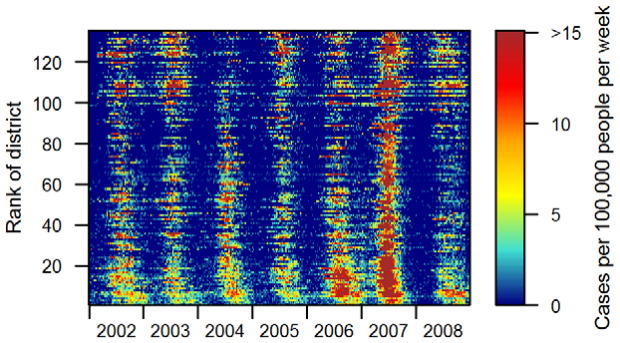
Apparent dengue haemorrhagic fever weekly incidence rates in the 135 most populous districts of Cambodia. Weekly incidence rates (cases per 100,000 people per week) were computed in each of the 135 districts where population density is higher than 20 people per km^2^ in Cambodia. Districts are ranked by increasing distance to Phnom Penh from bottom to top.

Figure 3 shows the weekly incidence in the 135 (/180) most populous districts (average district population of 78,000), where 105,598 of the 109,332 reported patients reside. Annual epidemics do not appear synchronous, peaking at different times of the year in different districts (between May and October). Unexpectedly, in Phnom Penh, the capital, where the virus circulates during the dry season [2], the annual epidemic lags behind the one in other districts indicating that Phnom Penh is not the starting point. Inspection of weekly and mean annual incidence maps over the whole period (not shown here, but partly visible in Figure 1, Movie S1, and Figure S1) showed two possible geographic axes seminal in the propagation of dengue: the national road between Kampong Cham and Siem Reap – the busiest one in Cambodia –, and the Mekong River.

To characterise and compare the spatio-temporal pattern of dengue incidence along those two axes and determine the focal starting areas of the annual epidemic, we performed a phase analysis using a wavelet approach (see Methods).

The examination of wavelet power spectra (Figure S2) revealed the annual seasonal component of incidence is the most powerful in 117/135 districts, meaning that in Cambodia, the seasonal cycle of dengue incidence time series has more power than the inter-annual cycle. A significant 2–3 years periodic component was detected in some districts, but our time series were too short in time to study it.

**Figure 4:**
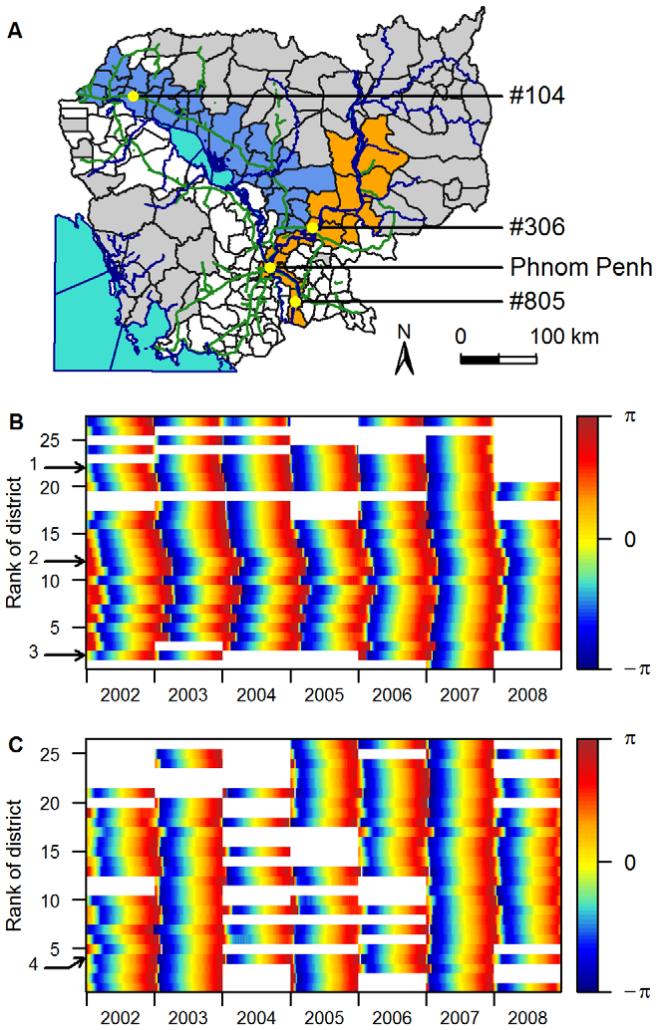
Phases of the annual component of incidence for districts located along two geographic axes. Phases are computed in the 0.8–1.2 year periodic band. (A) Map of the two geographic areas chosen: the national road in blue, and the Mekong River in orange. (B) Phase of districts along the Mekong River (orange in Figure 4A), presented from the most southerly to the most northerly from bottom to top. (C) Phase of districts along the national road (blue in Figure 4A), presented from West to East from bottom to top. The arrows indicate districts: 1, #306; 2, Phnom Penh; 3, #805 (Figure 4B); 4, #104 (Figure 4C).

Figure 4 represents, in a time-space domain, the phase of the annual component of incidence filtered in the 0.8–1.2 year periodic band for each district of the two geographic features identified previously (see Movie S2 for maps of these phases in all 135 districts). White parts represent years when no epidemic was detected in the district (see Methods). The phase represents the timing of the epidemic in each district. For a given week, if all districts have the same phase (same colour), the epidemics occur at the same time.

First, the pattern of spatial synchronicity observed is consistent from year to year. To test this result, each year, we ranked the 135 districts according to their phase during the epidemic period (weeks 13 to 39), from the district where the epidemic has the most advanced phase to the one with the highest phase delay. We then performed a Spearman correlation test on these ranks, comparing ranks from year n with ranks of years n+1. Except in 2007, correlation coefficients were all higher than 0.38 and significant (all p-values<0.03), inferring that districts are affected in a similar chronological order year after year. In 2007, the order in which districts were affected by the epidemic was not significantly correlated to the order of the previous year.

This ranking also allowed us, each year, to identify districts where the annual epidemic appears early. We have identified three starting points, all located in rural areas: district #306 and a few rural districts around (district #306 being the most early of them), district #104 and 2 other districts around, and, some years, district #805, located along the Mekong River, South to Phnom Penh, along the Vietnamese border (see Figure 4). These districts are very similar to other rural districts included in the analysis, composed of a flat flood plain, with a mean population density of 155 people per km^2^. They are all located away from the three urban centres where sentinel hospitals involved in active surveillance are. They are consistently the same, year after year.

Secondly, Figure 4 demonstrates that the propagation of dengue is heterogeneous. Annual epidemics are highly synchronous along the national road linking Kampong Cham to Siem Reap whereas an oriented propagation emanates from a rural area located around Kampong Cham (district #306) and ends in Phnom Penh 11 weeks later (95% C.I. 4–18 weeks). This was obscured when districts were classified independently of the geographic area, taking into account only the distance to Phnom Penh (Figures S3, S4 and S5). Some years, another oriented propagation is seen from a rural area near the Vietnamese border (district #805) towards Phnom Penh (Figure 4B).

**Figure 5:**
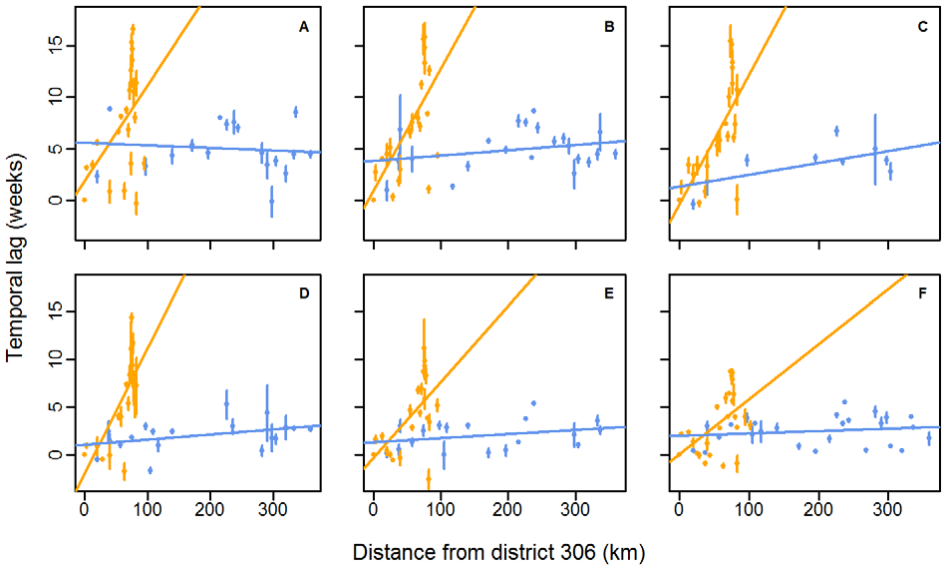
Scatterplot of mean annual temporal lags between epidemics against distances between districts. Temporal lags between epidemics and distances are computed relative to district #306. The lines show the linear regressions between the mean annual temporal lag of the annual epidemic in each district and the distance for 2002 (A), 2003 (B), 2004 (C), 2005 (D), 2006 (E) and 2007 (F). Colours represent the geographic localisation of each district, according to Figure 4A. The number of districts included in the analysis changes every year, according to whether an epidemic occurred in the district (Table 1). Error bars represent the 95% C.I. associated with the mean. Normality and homoscedasticity of residuals were confirmed using the Shapiro-Wilks and the Bartlett tests respectively (alpha level of 0.05).

To evaluate the speed of propagation along each axis, we calculated the temporal lag of the seasonal pattern in each district relative to the district #306 [10] (see Methods). This district was chosen as a time reference point to calculate time lags because it is located at the intersection of the Mekong River and the national road areas, which was convenient to present results of both geographic areas on the same figure (Figure 5). Moreover, based on the ranking of districts using their phase, district #306 was the one with the most number of districts lagging behind over the study period (132, 134, 131, 118, 124 and 104 districts/134 respectively from 2002 to 2007). The results of the analysis of covariance performed on the mean annual lag to compare the speed of propagation of the epidemic in each geographic axis (Figure 4A) are presented in Table 1. Results show that regardless of the year, the evolution of the temporal lag between epidemics according to the distance differs significantly (α-level of 0.05) from one area to the other. We therefore performed separate regressions for each of the two axes (Figure 5). Propagation is always faster along the national road than along the Mekong River. The speed of propagation of the travelling wave along the Mekong River, evaluated by the inverse of the regression slope, is estimated to be 11 km per week (95% C.I. 3–18 km per week) over the study period. Along the national road, the speed is quasi-instantaneous with respect to our time domain sampling rate.

**Table 1:** [Sec s3]Results of the analysis of covariance and linear regressions (see methods).

	Covariance analysis		Regression (“Mekong River”)			Regression (“national road”)	
year	n^1^	p-value of interaction	n^1^	p-value^2^	1/slope estimate^3^	n^1^	p-value^2^
2002	40	0.005	22	0.02	11	18	0.70
2003	44	<0.001	22	0.001	9	22	0.22
2004	31	<0.001	22	<0.001	8	9	0.07
2005	38	<0.001	19	<0.001	8	19	0.09
2006	40	<0.001	22	0.004	13	18	0.22
2007	52	0.001	26	0.006	17	26	0.378

1 Number of districts where an annual epidemic occurred according to the national threshold, along the Mekong River, the national road (Figure 4A), or, for the covariance analysis, both.

2 P-value of the regression slope estimate.

3 Inverse of the estimate of the regression slope β1, in km per week (see methods).

To test the role Phnom Penh plays in this heterogeneity, we ran the same analysis, but excluding Phnom Penh districts. The propagation remained significantly heterogeneous at an alpha-level of 5%, except in 2002 and 2007, and remained significantly heterogeneous at an alpha-level of 10% in 2002 and 2007 (results not shown).

The removal of districts with low population density has no effect at all on results shown. The exclusion of low incidence years did not modify the two conclusions arrived at in the paper (onset of the seasonal epidemic in highly localised rural areas, and heterogeneous propagation in the country), but removed the potential bias linked with the very low number of cases during the non epidemic years (see Figures S6 and S7 that show the same as Figures 4 and 5 when all years are included).

To sum up, our results show that the seasonal epidemic consistently starts in the same three rural districts in Cambodia. Then the propagation is not homogeneous in the country. In districts located along the busiest road, dengue epidemics appear simultaneously and early (with all districts being hit in less than a month), whereas districts located along the Mekong River get hit by the seasonal epidemic later, with the delay being proportional to the distance to district #306.

In 2007 an exceptional epidemic occurred in Cambodia with DENV-3 as the dominant etiological agent, and a four fold increase in weekly incidence rate compared to the four previous years (Figures 2 and 3). During this epidemic, the wave in the one-year periodic mode travelled from Kampong Cham to Phnom Penh in only 7 weeks, at a speed of 17 km per week, a higher synchronicity reflecting a more rapid propagation during this peculiar event. The sequence in which districts were affected by the seasonal variation was also modified, as shown by non-significant Spearman rank coefficients when correlations were calculated between any year and the year 2007.

## Discussion

### A rural onset

Regarding the onset of the national epidemic, there is a tendency for the dengue season to start in few rural districts more often than in any other district moving from rural areas towards urban centres, with, for example, annual epidemics in districts #306 and #104 (Figure 4A) leading epidemics in the rest of the country by 3 weeks on average over the study period (95% C.I. 2–4 weeks). In addition, recent prospective cohort data showed that rural areas were affected by dengue to the same degree as urban areas or, as during the 2007 epidemic, at even higher incidence rates [18]. This finding is not consistent with the common thought that the most populated areas spread the disease [7]–[9] or known mechanisms underlying travelling waves [7], [13]. One plausible explanation for a rural origin of the spread, also supported by a recent study in Vietnam [19], is that more than 80% of rural Cambodians do not have access to public water supply, and store their drinking water in big jars that have been identified as major breeding sites for *Aedes* mosquitoes in Cambodia [2]. This result (onset in rural areas) has strong implications regarding the control of dengue in low-income countries.

### Factors that could explain the heterogeneous pattern of propagation

To our knowledge, it is the first time that a recurrent heterogeneous pattern of propagation of dengue is revealed in surveillance data at a national scale. This pattern of propagation could come from actual transmission of dengue viruses between districts, via infected mosquitoes or humans, or correspond merely to spatial differences in the emergence of the epidemic, differences due either to spatial differences in local (within-district) dynamics, or to forcing by extrinsic factors such as climate (Moran effect). Given the limited dispersal range of *Aedes spp.* vectors and the high synchronicity of the epidemics over a 400 km wide area along the national road, it is unlikely that viral transmission from one place to another via infected mosquitoes can account for the pattern observed.

Climate is quite homogeneous in Cambodia, with a narrow temperature range across the country, and the monsoon striking the country from South to North within a month only, around April-May. Climate, by influencing the vector's life cycle, is clearly driving the dengue seasonality observed in Cambodia. It could easily explain the high synchronicity of epidemics observed along the national road if this synchronisation was observed country-wide. However, two of our observations preclude climatic forcing to be the underlying factor for the spatial-temporal pattern observed. First, climate is more homogeneous in the country compared to the observed spatial-temporal pattern of dengue fever. The existence of a very slow oriented dengue propagation along the Mekong River, going North to South, with the epidemic in some districts lagging more than 3 months behind the one in districts that are only less than 200 kilometres in distance cannot be accounted for by climatic differences between districts. Secondly, the epidemic starts as early as in March in the three districts identified as starting points, whereas the monsoon only begins end of April/beginning of May.

We believe the heterogeneous propagation observed is related to the heterogeneity of traffic on the roads of the country, where traffic allows the movement of human carriers or transported mosquitoes infected with dengue. The national road –the busiest country road connecting the two main economic cities within 4–5 hours – probably explains synchronicity. By contrast, the existence of dirt roads along the entire Mekong River, on which traffic and population movement between smaller villages are slower and more difficult than along the national road supports our hypothesis. Despite its epidemiological relevance, our understanding of the relationship between human movement and pathogen transmission remains limited [20]–[22]. The importance of the relationship between human movement and pathogen transmission in explaining the spatio-temporal dynamics of dengue incidence or other diseases has been increasingly pointed-out during the last decade [5], [8], [9],[23]–[25] and explored mainly through a theoretical modelling approach [22], [26], [27].

### 2007, a peculiar year

The higher synchronisation and higher incidence levels across the country in 2007 argues for an association with higher net reproduction ratios of infection due to lower herd immunity when a new serotype is introduced [25], [28]. This hypothesis is supported by the fact that serotype 3 invaded Cambodia at the end of year 2006, replacing serotype 2, in place since 1999, as the major circulating virus [2]. The fact that heterogeneity of propagation remained despite this serotype change supports the hypothesis that another factor – such as human movement – plays an important role in the dynamics.

### Limitations

Major limitations of our analysis include underreporting inherent to passive surveillance data and potential selection bias leaning towards underreporting from urban centres compared with rural areas. However, underreporting would only affect the amplitude of the epidemics in each district and therefore have little effects on the study of synchronism when using the wavelet phase analysis approach [12]. Secondly, it is unlikely that rural Cambodians were over-represented as most hospitals and those that recruit dengue patients through active surveillance are free of charge and located in urban areas (three cities). One could also assume that collecting data from both an active and a passive surveillance system could affect the timing of detection of the epidemics, with active surveillance sites more likely to reveal a small increase in incidence levels earlier than passive surveillance sites. This would lead to a bias in the analysis. However, after a thorough analysis of the surveillance system (Institut Pasteur Cambodia's not published report), we believe that severe cases are scarcely missed even by the passive surveillance system; we also believe that our results are not affected by this bias, given the fact that districts affected early in the epidemic are located in rural areas, kilometres away from the urban sentinel sites. Another common limitation when analysing surveillance data is the introduction of spatial or temporal biases due to difficulties in standardising surveillance systems in time and space, especially in low income countries. We thus, on purpose, excluded any results or comments that would rely on spatial differences in incidence levels only. We found the wavelet approach very robust to these biases when exploring spatial-temporal patterns: unlike many other temporal methods, the level of incidence in a given district does not influence the calculation of time lags between epidemics.

One could think that not standardising incidence according to age could impair the results obtained. But age standardisation only affects the results by modifying incidence levels, and as our results do not rely on differences in incidence levels, this is not a real concern.

### Perspectives

Our findings and speculations require further research for additional evidence. Firstly, reasons as to why the three areas identified as being hit early by the epidemic repeatedly year after year (districts #306, #104 and #805) are unclear. This warrants further filed investigations to identify specific factors that trigger the epidemic in these settings.

Secondly, the data we analysed here can only reveal the spatio-temporal dynamics and help make hypotheses on underlying factors. Given the results of this study, and particularly the heterogeneity of dengue spatial dynamics, surveillance data on the spatial distribution of the serotypes (and genotypes if possible) of the co-circulating dengue viruses would help validate (or not) our hypotheses on dengue dynamics in Cambodia, using independent data.

Lastly, given the potential benefit in term of disease control from demonstrating the efficient role of humans' movement in dengue spatial transmission, one might consider further investigations in this direction, collecting data and using dynamic models for instance.

## Supporting Information

Figure S1 **Maps of weekly incidence rates in Cambodia.** Maps show weeks 5, 9, 11, 14, 18, 22, 25, 28, 31, 35, 38, 42 and 50 of year 2002 in Cambodian districts (in number of cases declared per 100,000 people per week).
(PDF)

Figure S2 **Map of Cambodian districts with their identifying numbers and wavelet power spectra of the weekly incidence rates of districts with more than 20 people per km^2^.** Power is colour coded, blue indicating low power. Dashed lines represent the limit of the cone of influence where data are modified by edge effects. The black lines show the 0.05 alpha-level of significance computed based on 500 bootstrapped series [10].
(PDF)

Figure S3 **Phases of the annual component of incidence for districts located along the “Mekong” axis (orange in** **figure 4A** **).** Phases are computed in the 0.8–1.2 year periodic band. Districts are ranked by increasing distance to Phnom Penh, from bottom to top. The arrows indicate: 1, district #306; 2, Phnom Penh; 3, district #805.
(TIF)

Figure S4 **Phases of the annual component of incidence for districts located along the “national road” axis (blue in** **figure 4A** **).** Phases are computed in the 0.8–1.2 year periodic band. Districts are ranked by increasing distance to Phnom Penh, from bottom to top. The arrow indicates district #104.
(TIF)

Figure S5 **Phases of the annual component of incidence for districts with more than 20 people per km^2^.** Phases are computed in the 0.8–1.2 year periodic band. Districts are ranked by increasing distance to Phnom Penh, from bottom to top. The arrows indicate: 1, Phnom Penh; 2, District #306; 3, district #805; 4, district #104 (see figure 4A).
(TIF)

Figure S6 **Phases of the annual component of incidence for districts located along two geographic axes (see** **Figure 4A** **for a map of the two geographic areas chosen).** Phases are computed in the 0.8–1.2 year periodic band. Figure shows the same as Figure 4B and 4C, but with all years included. (A) Phase of districts along the Mekong River (orange in Figure 4A), presented from the most southerly to the most northerly from bottom to top. (B) Phase of districts along the national road (blue in Figure 4A), presented from West to East from bottom to top. The arrows indicate districts: 1, #306; 2, Phnom Penh (Figure S6A) and #104 (Figure S6B).
(TIF)

Figure S7 **Scatterplot of mean annual temporal lags between epidemics against distances between districts.** Temporal lags between epidemics and distances are computed relative to district #306. The lines show the linear regressions between the mean annual temporal lag of the annual epidemic in each district and the distance for 2002 (A), 2003 (B), 2004 (C), 2005 (D), 2006 (E) and 2007 (F). Colours represent the geographic localisation of each district, according to Figure 4A. Error bars represent the 95% C.I. associated with the mean. Figure shows the same as Figure 5, but with all years included. Each year, the number of units included in the analysis is 27 for the Mekong axis, and 26 for the national road axis.
(TIF)

Movie S1 **Evolution of weekly incidence rates in Cambodia.** Incidence rates are shown on maps of Cambodian districts from January 2002 to December 2008.
(MOV)

Movie S2 **Evolution the weekly phases of the annual component of incidence.** Phases are computed in the 0.8–1.2 year periodic band and shown on maps of Cambodian districts from January 2002 to December 2008.
(MOV)
