# Supplementary figures and images for: Can Human Movements Explain Heterogeneous Propagation of Dengue Fever in Cambodia?
Source: PLoS Negl Trop Dis. 2012 Dec 6;6(12):e1957. doi: 10.1371/journal.pntd.0001957 (PMC3516584; doi:10.1371/journal.pntd.0001957)

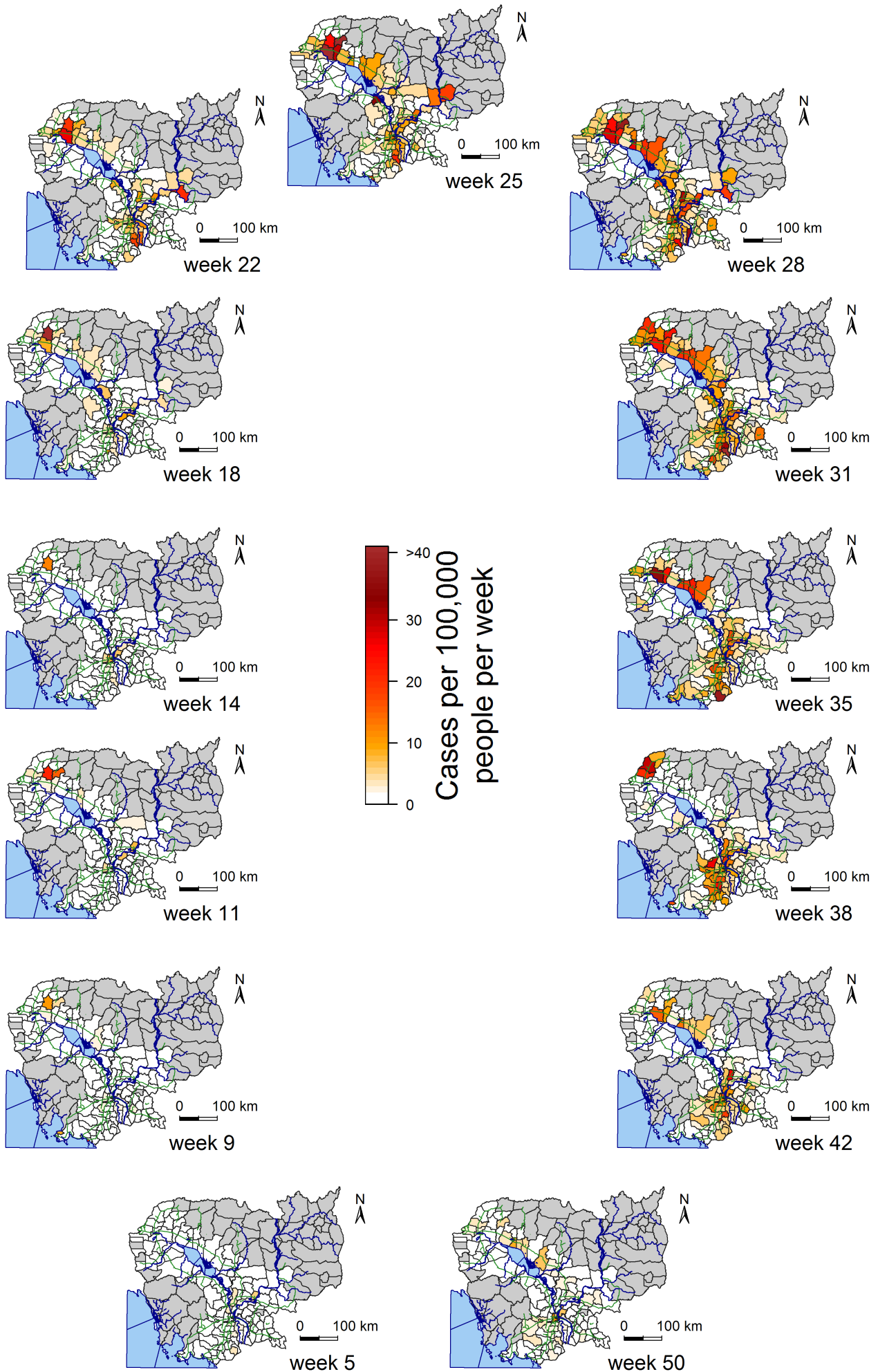

Supplement: Figure S1 — Maps of weekly incidence rates in Cambodia. Maps show weeks 5, 9, 11, 14, 18, 22, 25, 28, 31, 35, 38, 42 and 50 of year 2002 in Cambodian districts (in number of cases declared per 100,000 people per week). (PDF) [file pntd.0001957.s001.pdf]

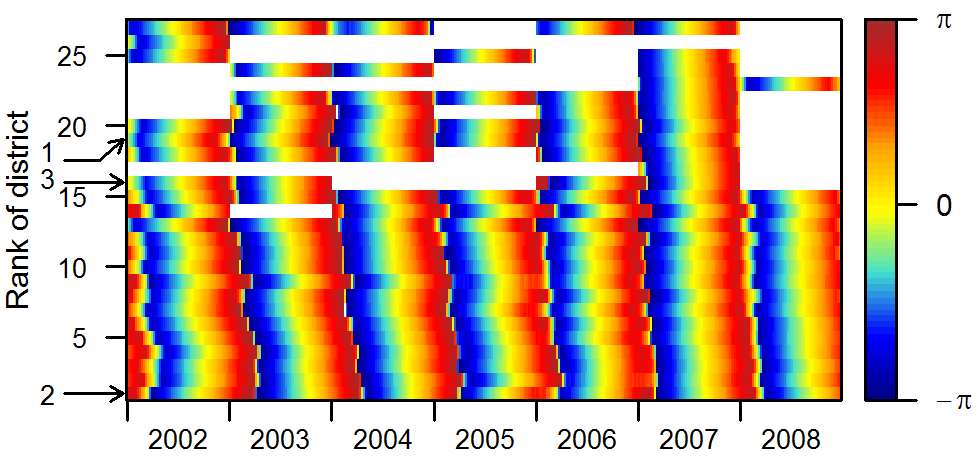

Supplement: Figure S3 — Phases of the annual component of incidence for districts located along the “Mekong” axis (orange in figure 4A ). Phases are computed in the 0.8–1.2 year periodic band. Districts are ranked by increasing distance to Phnom Penh, from bottom to top. The arrows indicate: 1, district #306; 2, Phnom Penh; 3, district #805. (TIF) [file pntd.0001957.s003.tif]

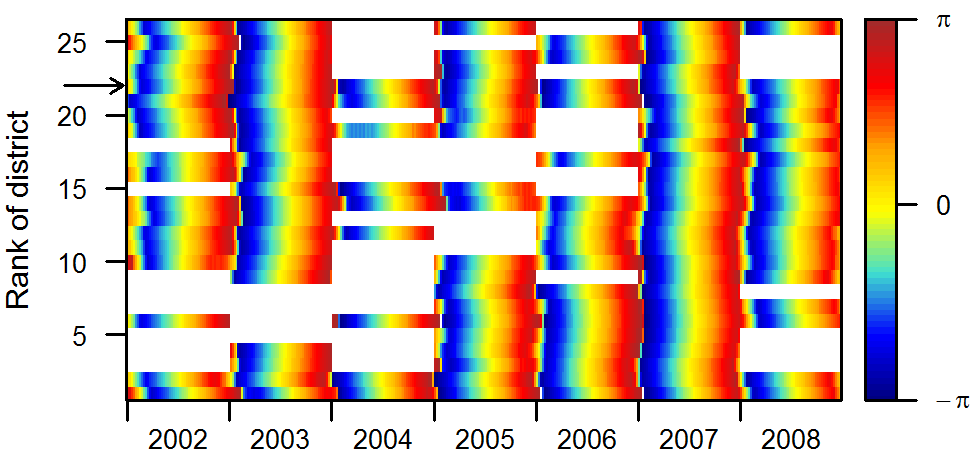

Supplement: Figure S4 — Phases of the annual component of incidence for districts located along the “national road” axis (blue in figure 4A ). Phases are computed in the 0.8–1.2 year periodic band. Districts are ranked by increasing distance to Phnom Penh, from bottom to top. The arrow indicates district #104. (TIF) [file pntd.0001957.s004.tif]

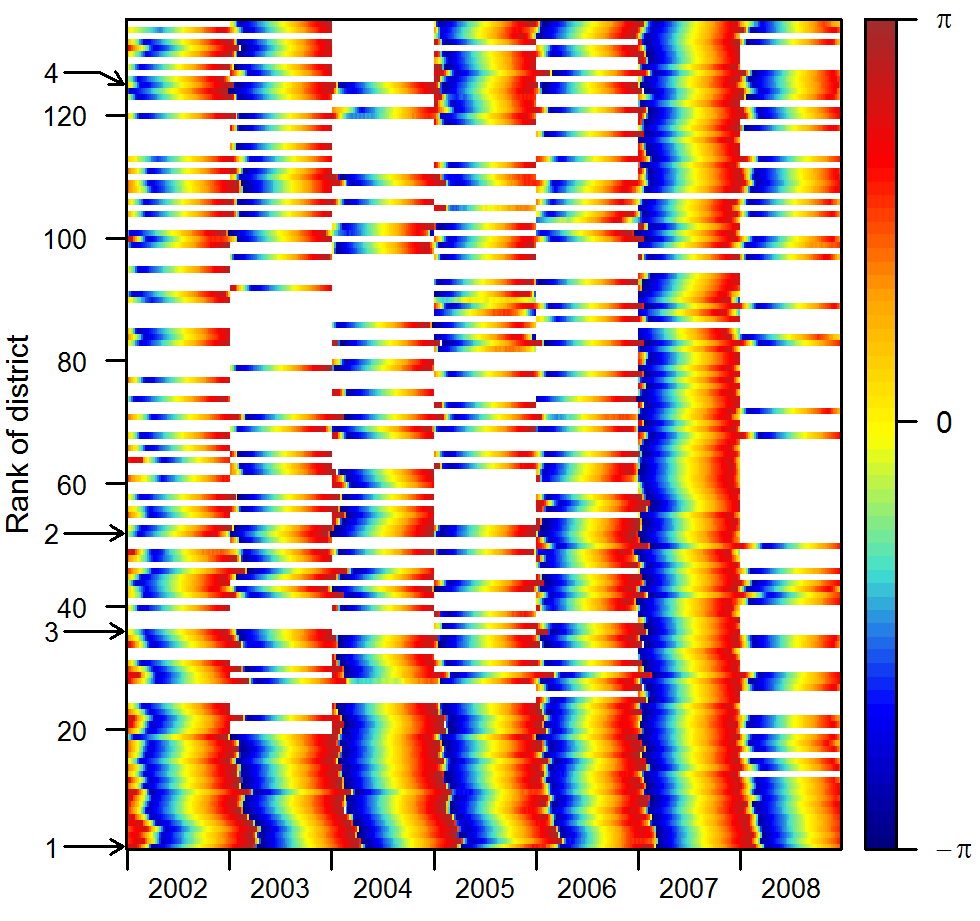

Supplement: Figure S5 — Phases of the annual component of incidence for districts with more than 20 people per km2. Phases are computed in the 0.8–1.2 year periodic band. Districts are ranked by increasing distance to Phnom Penh, from bottom to top. The arrows indicate: 1, Phnom Penh; 2, District #306; 3, district #805; 4, district #104 (see figure 4A). (TIF) [file pntd.0001957.s005.tif]

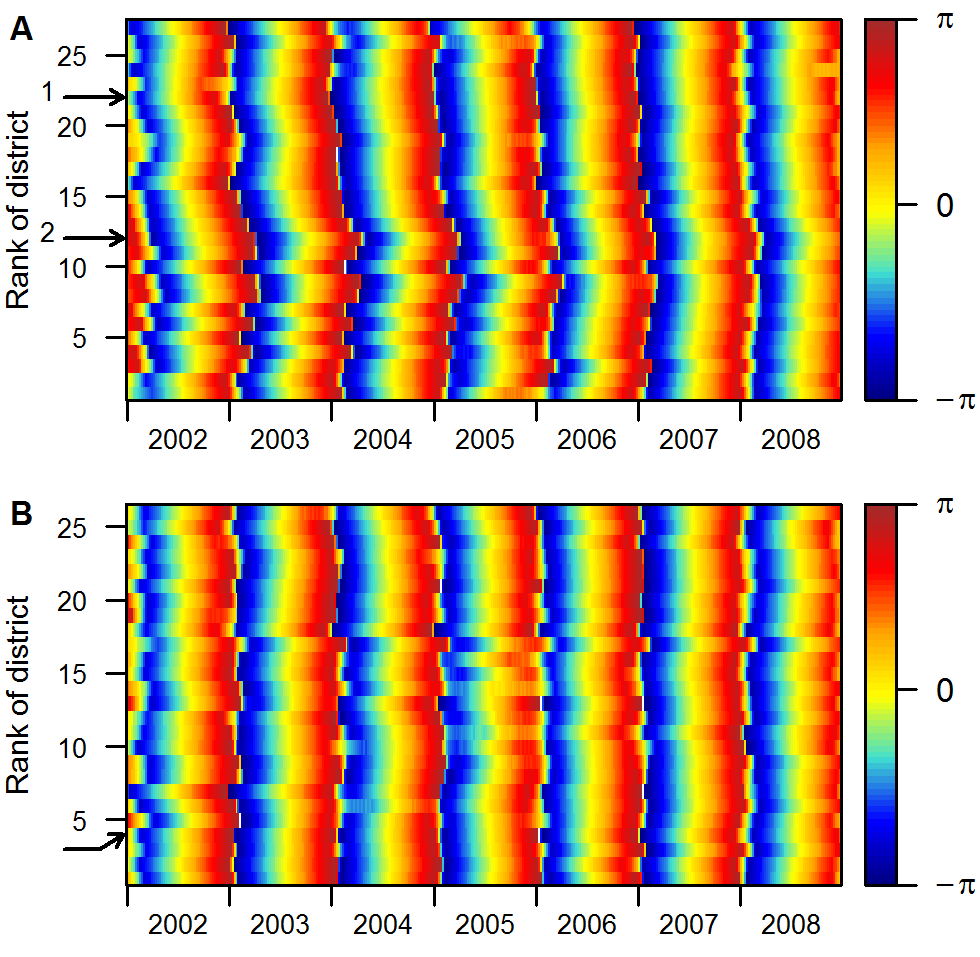

Supplement: Figure S6 — Phases of the annual component of incidence for districts located along two geographic axes (see Figure 4A for a map of the two geographic areas chosen). Phases are computed in the 0.8–1.2 year periodic band. Figure shows the same as Figure 4B and 4C, but with all years included. (A) Phase of districts along the Mekong River (orange in Figure 4A), presented from the most southerly to the most northerly from bottom to top. (B) Phase of districts along the national road (blue in Figure 4A), presented from West to East from bottom to top. The arrows indicate districts: 1, #306; 2, Phnom Penh (Figure S6A) and #104 (Figure S6B). (TIF) [file pntd.0001957.s006.tif]

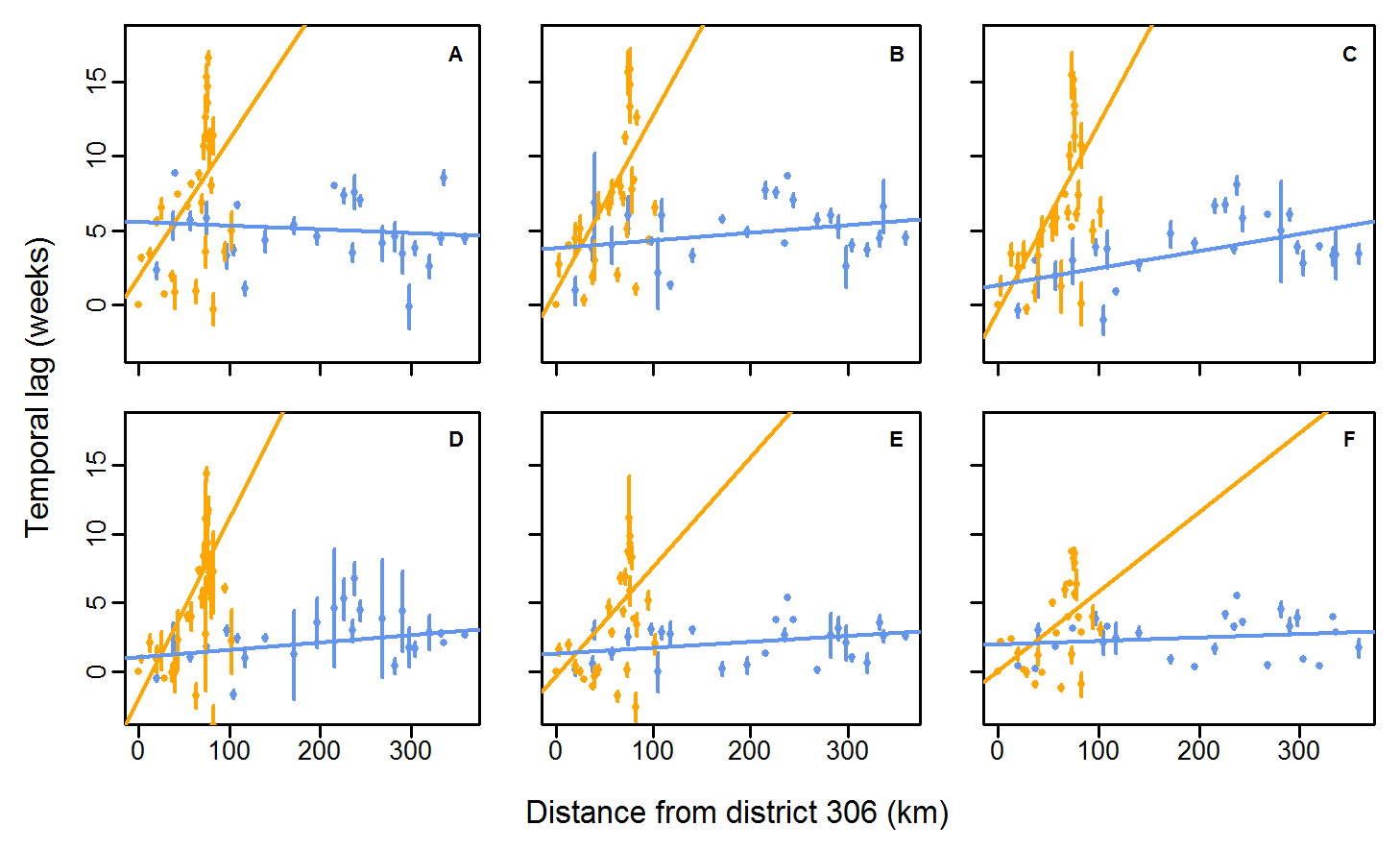

Supplement: Figure S7 — Scatterplot of mean annual temporal lags between epidemics against distances between districts. Temporal lags between epidemics and distances are computed relative to district #306. The lines show the linear regressions between the mean annual temporal lag of the annual epidemic in each district and the distance for 2002 (A), 2003 (B), 2004 (C), 2005 (D), 2006 (E) and 2007 (F). Colours represent the geographic localisation of each district, according to Figure 4A. Error bars represent the 95% C.I. associated with the mean. Figure shows the same as Figure 5, but with all years included. Each year, the number of units included in the analysis is 27 for the Mekong axis, and 26 for the national road axis. (TIF) [file pntd.0001957.s007.tif]
