# Supplementary material for: Can Human Movements Explain Heterogeneous Propagation of Dengue Fever in Cambodia?
Source: PLoS Negl Trop Dis. 2012 Dec 6;6(12):e1957. doi: 10.1371/journal.pntd.0001957 (PMC3516584; doi:10.1371/journal.pntd.0001957)
Supplement: Figure S2 — Map of Cambodian districts with their identifying numbers and wavelet power spectra of the weekly incidence rates of districts with more than 20 people per km2. Power is colour coded, blue indicating low power. Dashed lines represent the limit of the cone of influence where data are modified by edge effects. The black lines show the 0.05 alpha-level of significance computed based on 500 bootstrapped series [10]. (PDF) [file pntd.0001957.s002.pdf]

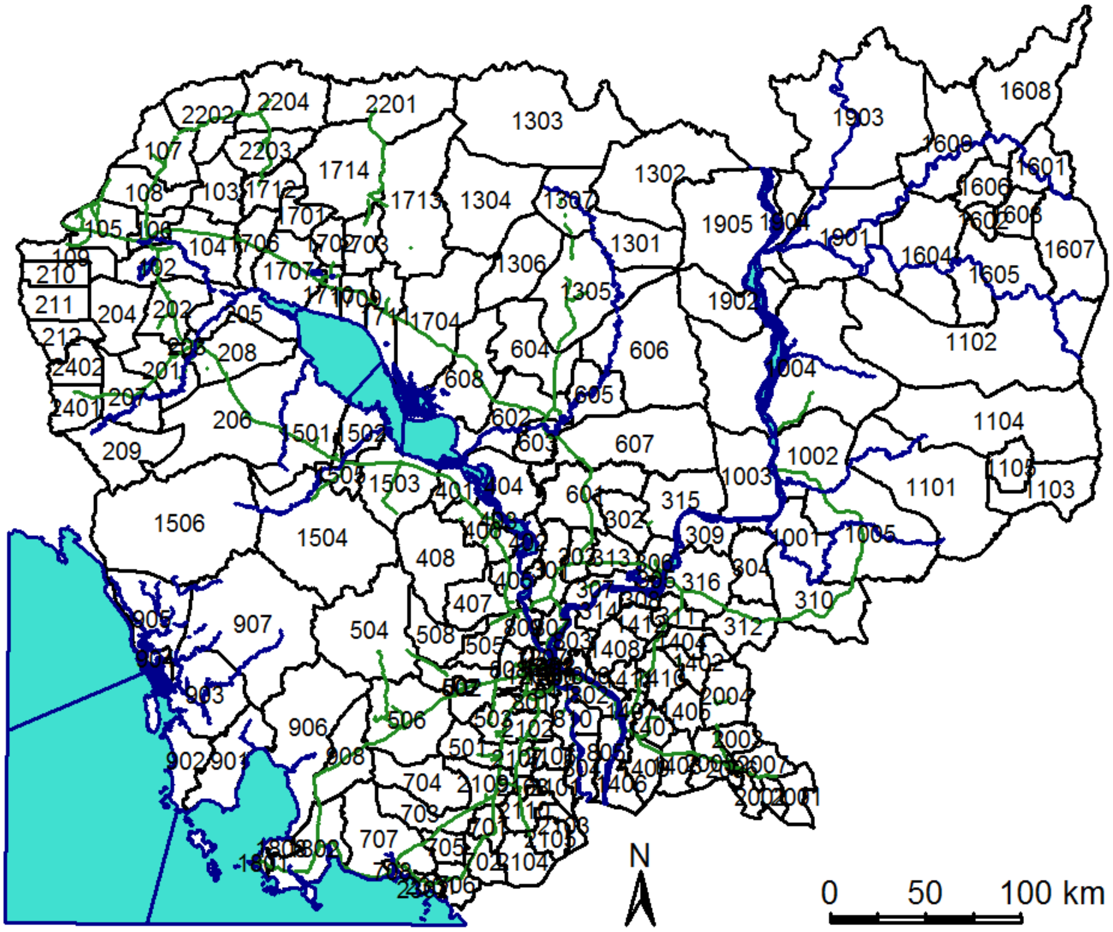
N
0 50 100 km

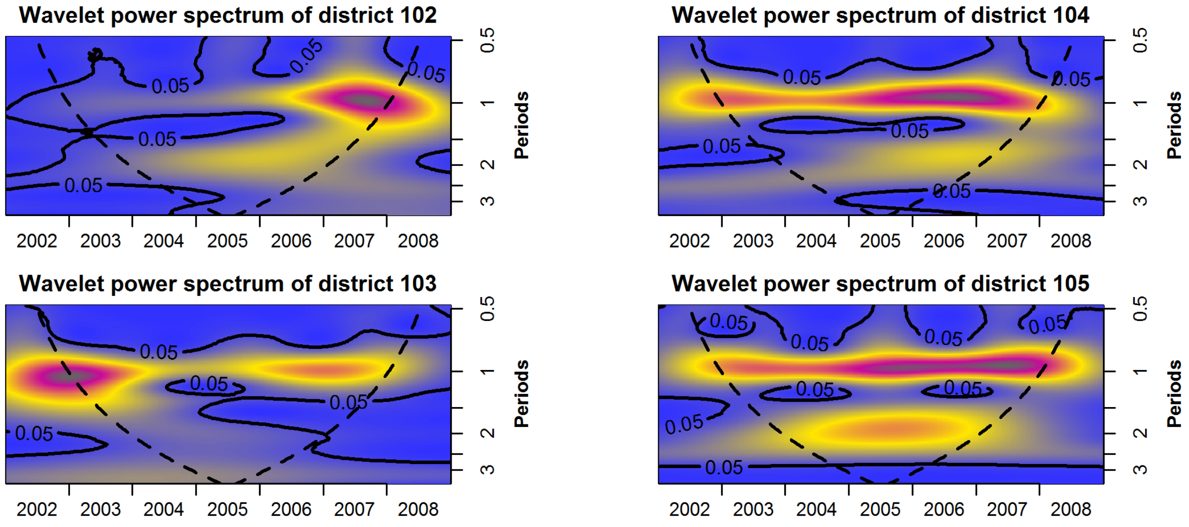
Wavelet power spectrum of district 102
Wavelet power spectrum of district 104
Wavelet power spectrum of district 103
Wavelet power spectrum of district 105
Periods
0.05
2002 2003 2004 2005 2006 2007 2008

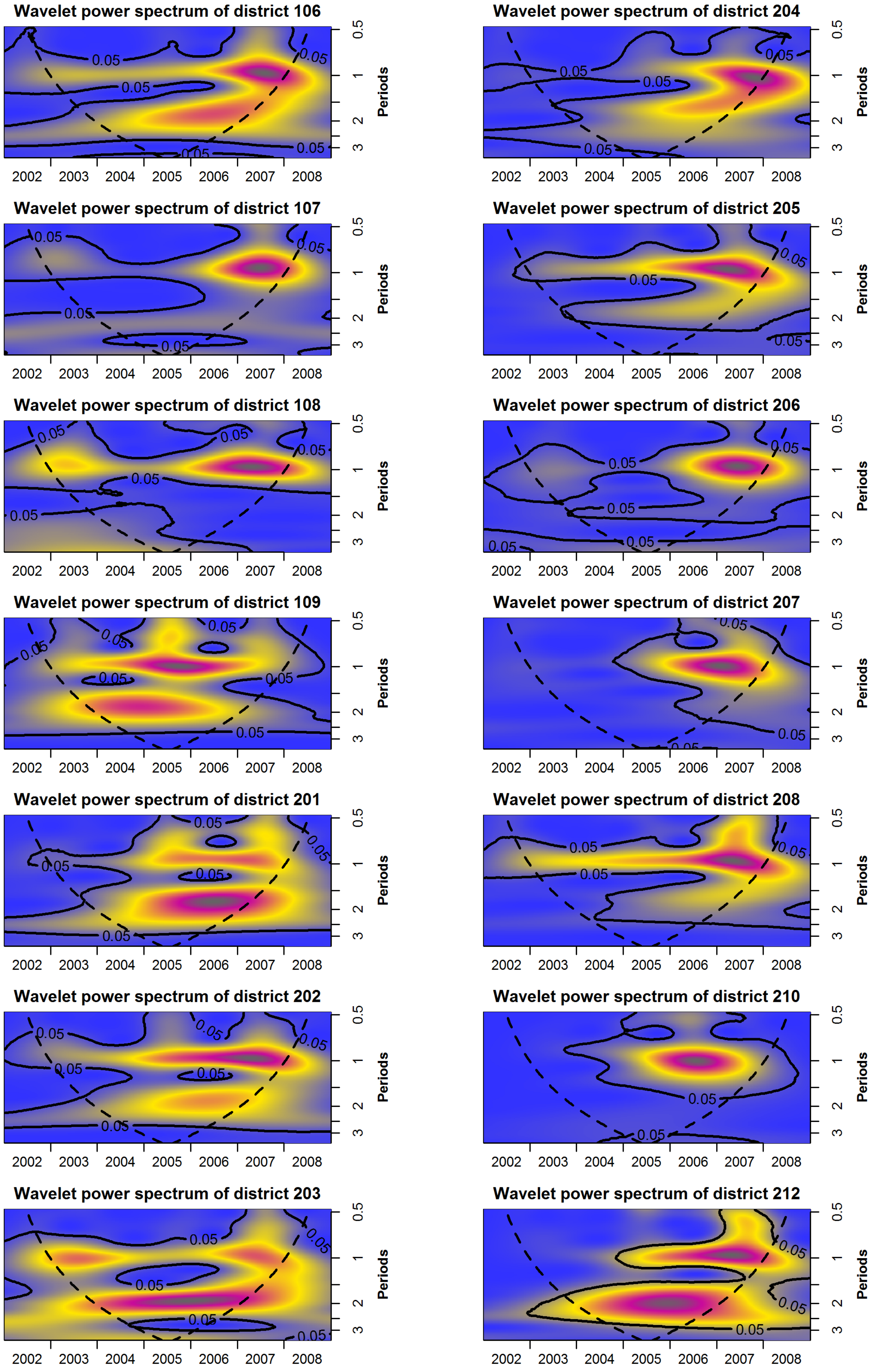
Wavelet power spectrum of district 106
Wavelet power spectrum of district 204
Wavelet power spectrum of district 107
Wavelet power spectrum of district 205
Wavelet power spectrum of district 108
Wavelet power spectrum of district 206
Wavelet power spectrum of district 109
Wavelet power spectrum of district 207
Wavelet power spectrum of district 201
Wavelet power spectrum of district 208
Wavelet power spectrum of district 202
Wavelet power spectrum of district 210
Wavelet power spectrum of district 203
Wavelet power spectrum of district 212
Periods
0.5
1
2
3
2002
2003
2004
2005
2006
2007
2008
0.05

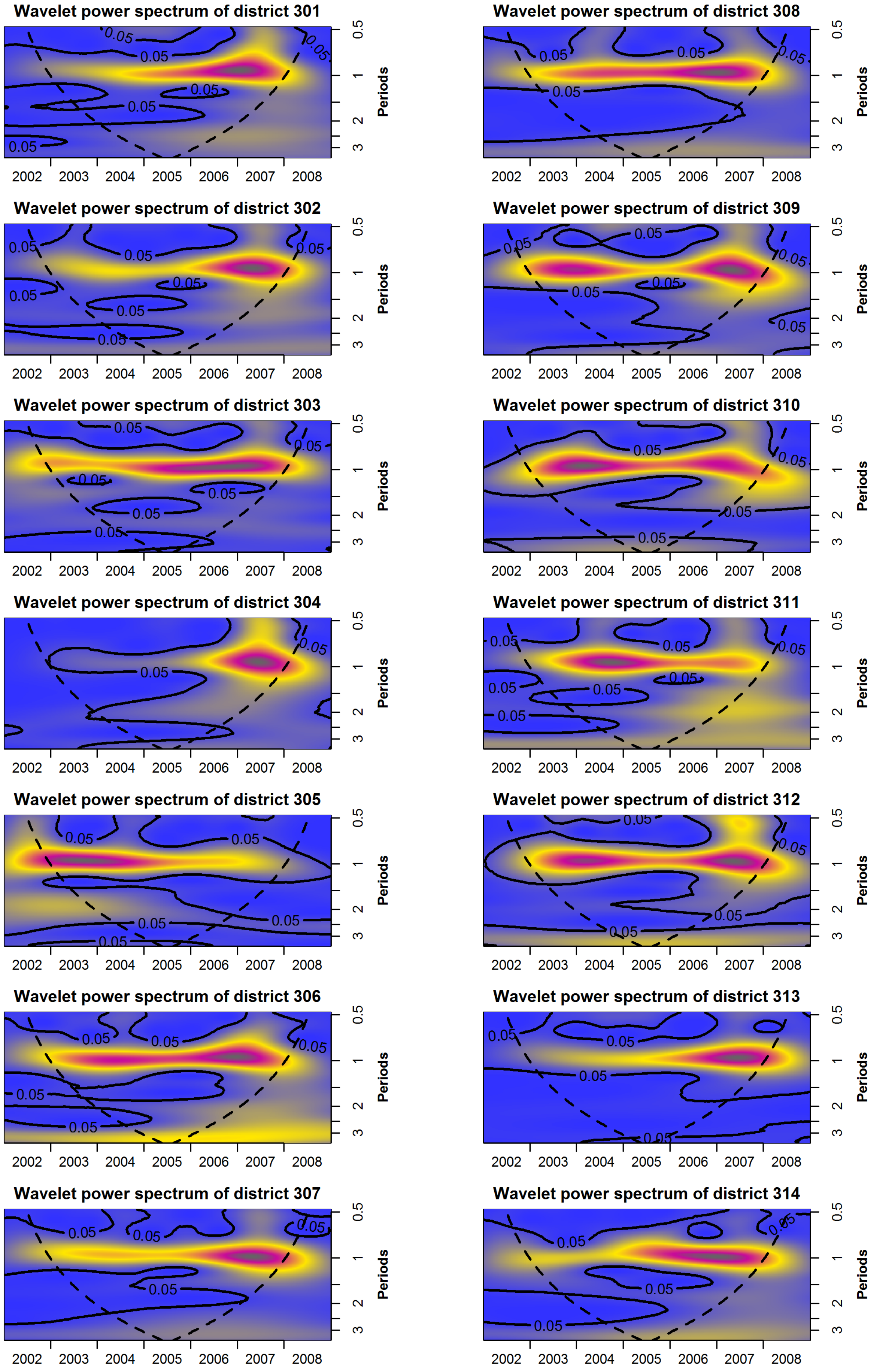

Wavelet power spectrum of district 301
Wavelet power spectrum of district 302
Wavelet power spectrum of district 303
Wavelet power spectrum of district 304
Wavelet power spectrum of district 305
Wavelet power spectrum of district 306
Wavelet power spectrum of district 307
Wavelet power spectrum of district 308
Wavelet power spectrum of district 309
Wavelet power spectrum of district 310
Wavelet power spectrum of district 311
Wavelet power spectrum of district 312
Wavelet power spectrum of district 313
Wavelet power spectrum of district 314
Periods
0.5
1
2
3
2002
2003
2004
2005
2006
2007
2008

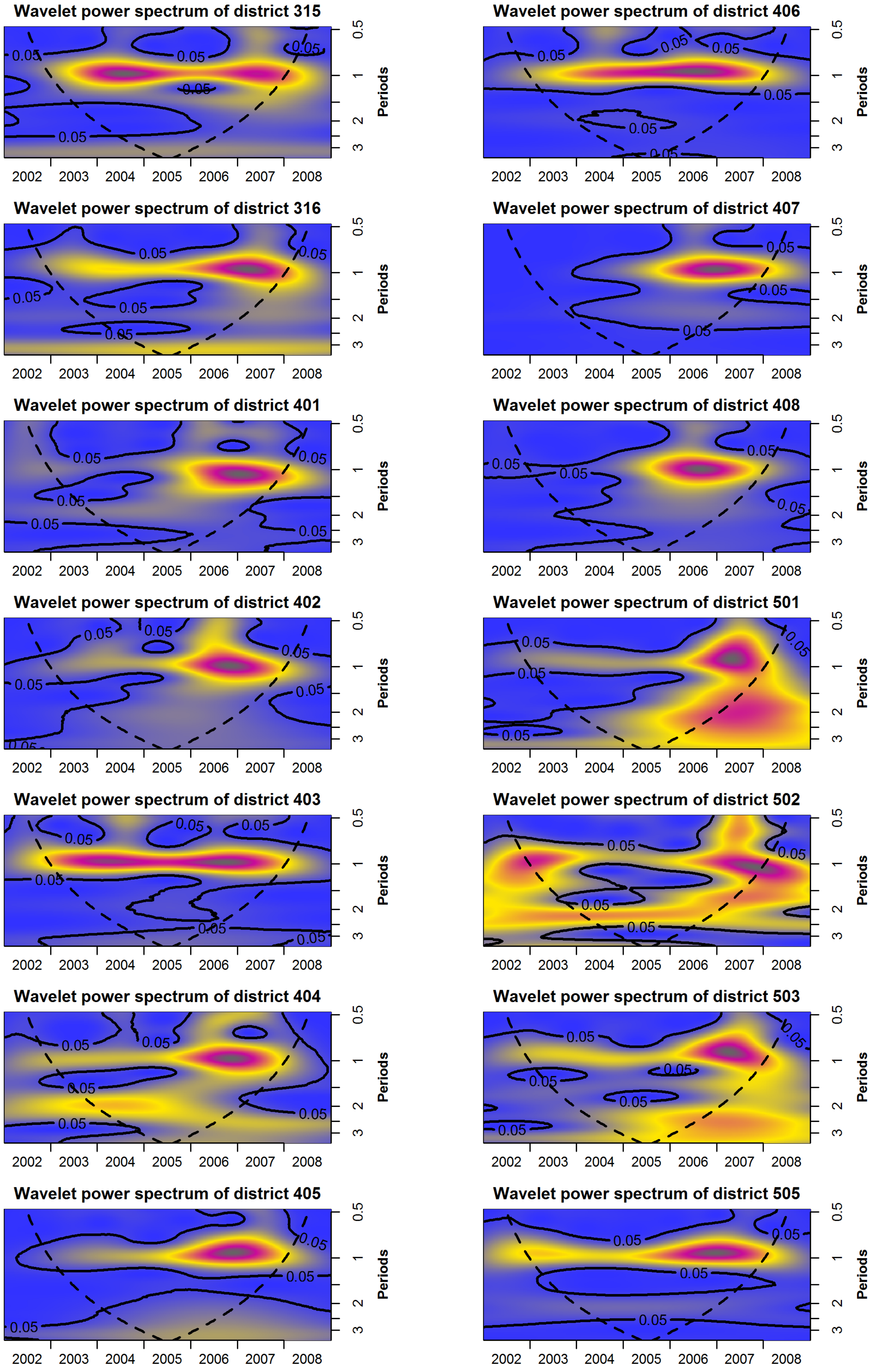
Wavelet power spectrum of district 315
Wavelet power spectrum of district 406
Wavelet power spectrum of district 316
Wavelet power spectrum of district 407
Wavelet power spectrum of district 401
Wavelet power spectrum of district 408
Wavelet power spectrum of district 402
Wavelet power spectrum of district 501
Wavelet power spectrum of district 403
Wavelet power spectrum of district 502
Wavelet power spectrum of district 404
Wavelet power spectrum of district 503
Wavelet power spectrum of district 405
Wavelet power spectrum of district 505
2002 2003 2004 2005 2006 2007 2008
0.5
1
2
3
Periods
0.05

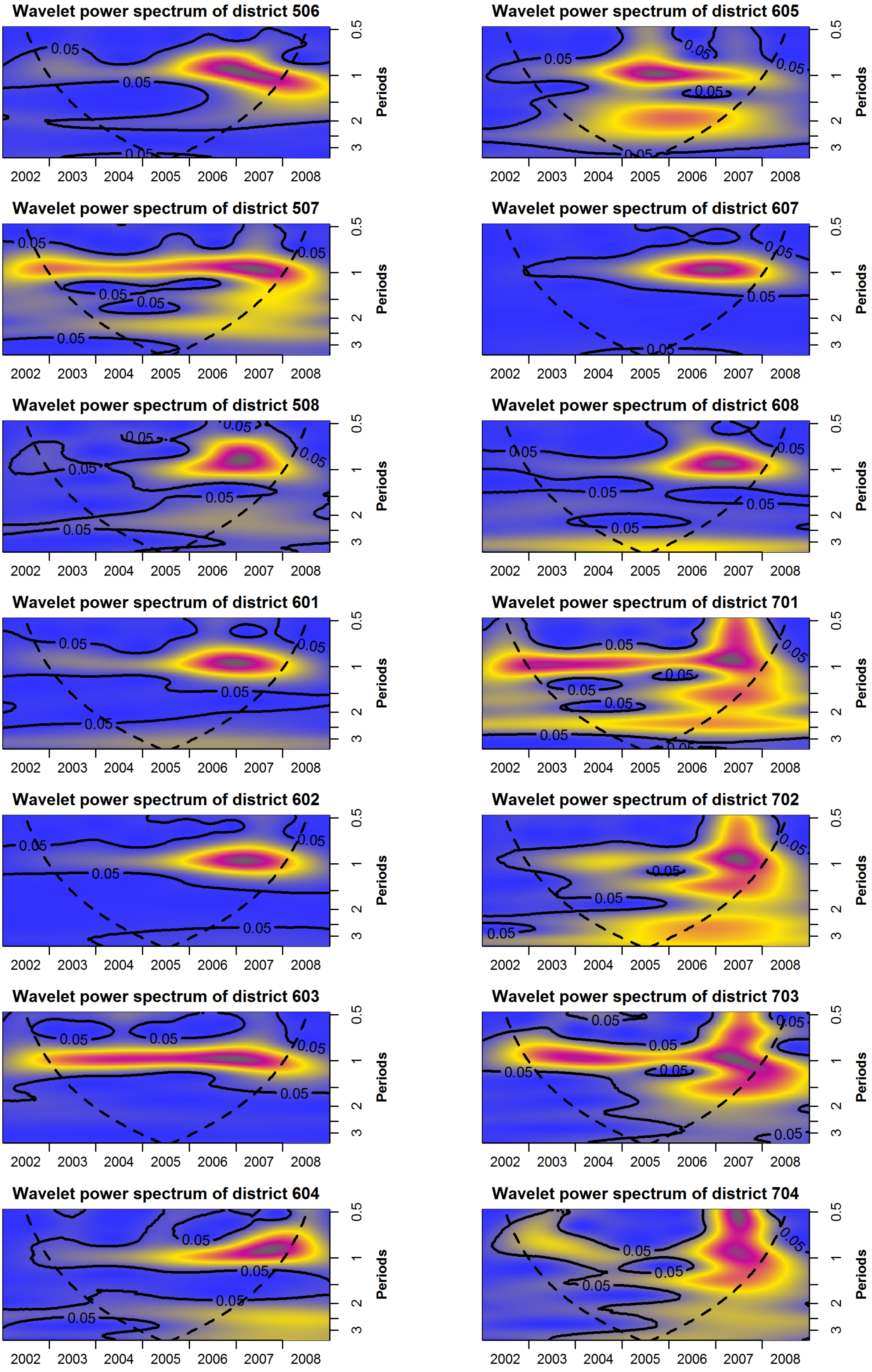
Wavelet power spectrum of district 506
Wavelet power spectrum of district 605
Wavelet power spectrum of district 507
Wavelet power spectrum of district 607
Wavelet power spectrum of district 508
Wavelet power spectrum of district 608
Wavelet power spectrum of district 601
Wavelet power spectrum of district 701
Wavelet power spectrum of district 602
Wavelet power spectrum of district 702
Wavelet power spectrum of district 603
Wavelet power spectrum of district 703
Wavelet power spectrum of district 604
Wavelet power spectrum of district 704
2002 2003 2004 2005 2006 2007 2008
Periods
0.5 1 2 3
0.05

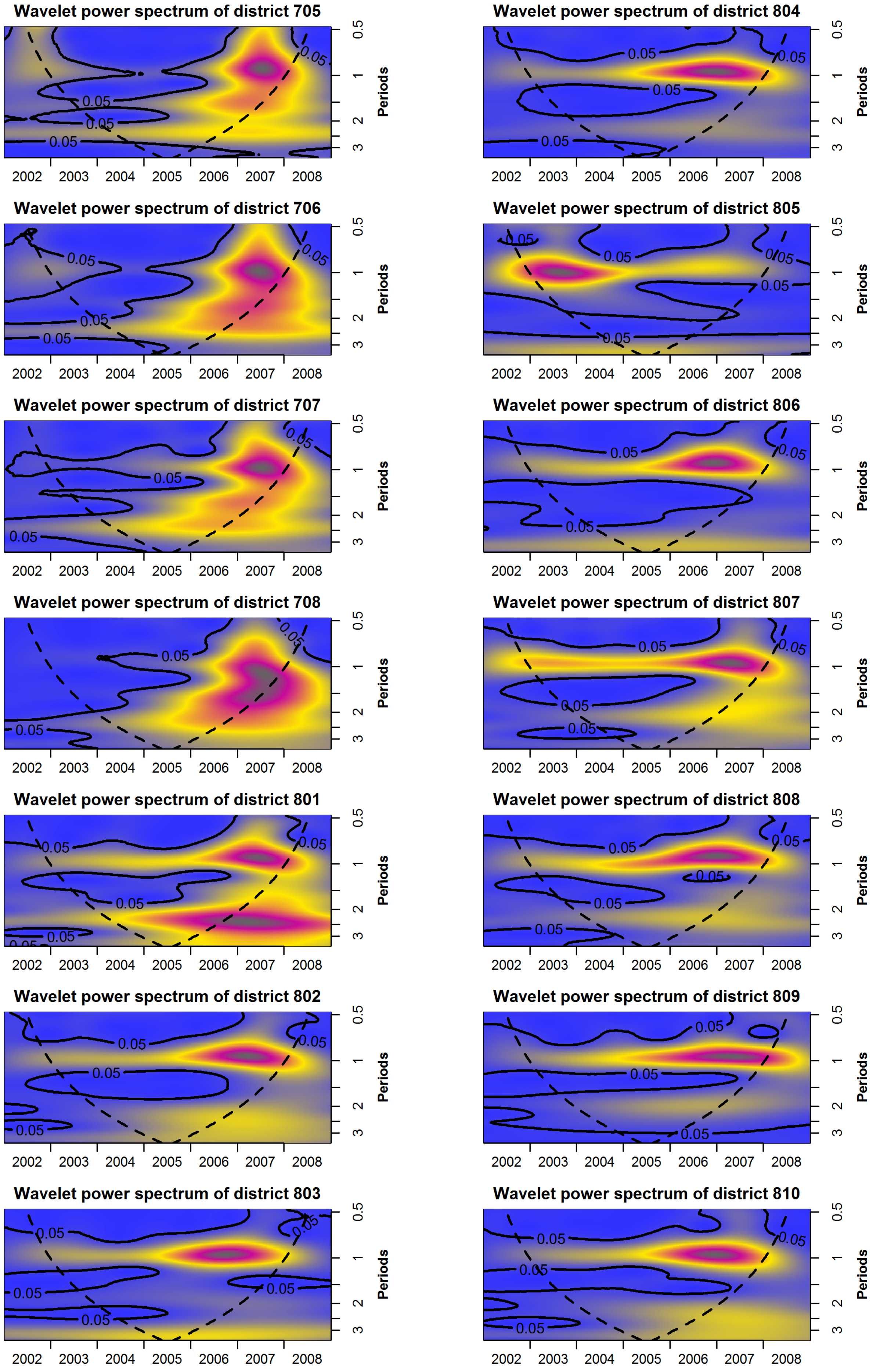
Wavelet power spectrum of district 705
Wavelet power spectrum of district 804
Wavelet power spectrum of district 706
Wavelet power spectrum of district 805
Wavelet power spectrum of district 707
Wavelet power spectrum of district 806
Wavelet power spectrum of district 708
Wavelet power spectrum of district 807
Wavelet power spectrum of district 801
Wavelet power spectrum of district 808
Wavelet power spectrum of district 802
Wavelet power spectrum of district 809
Wavelet power spectrum of district 803
Wavelet power spectrum of district 810
Periods
0.5
1
2
3
2002
2003
2004
2005
2006
2007
2008
0.05

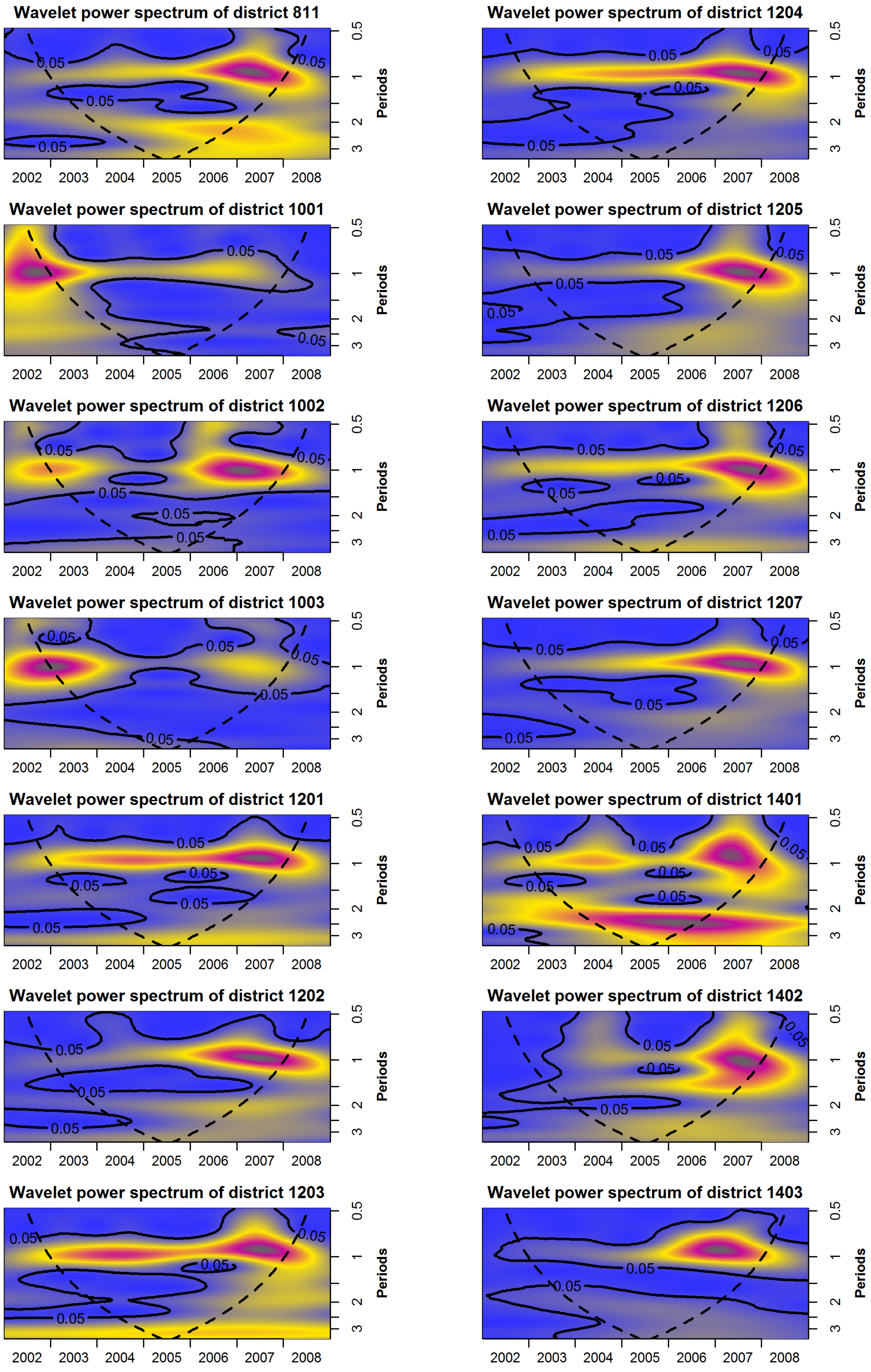
Wavelet power spectrum of district 811
Wavelet power spectrum of district 1204
Wavelet power spectrum of district 1001
Wavelet power spectrum of district 1205
Wavelet power spectrum of district 1002
Wavelet power spectrum of district 1206
Wavelet power spectrum of district 1003
Wavelet power spectrum of district 1207
Wavelet power spectrum of district 1201
Wavelet power spectrum of district 1401
Wavelet power spectrum of district 1202
Wavelet power spectrum of district 1402
Wavelet power spectrum of district 1203
Wavelet power spectrum of district 1403
Periods
0.5
1
2
3
2002
2003
2004
2005
2006
2007
2008
0.05

Wavelet power spectrum of district 1405

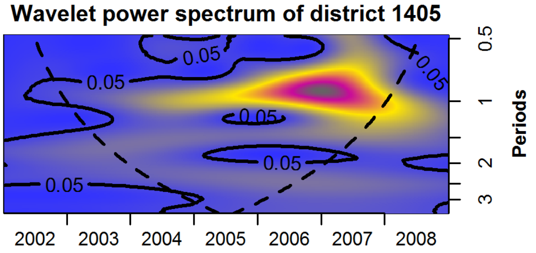

Wavelet power spectrum of district 1406

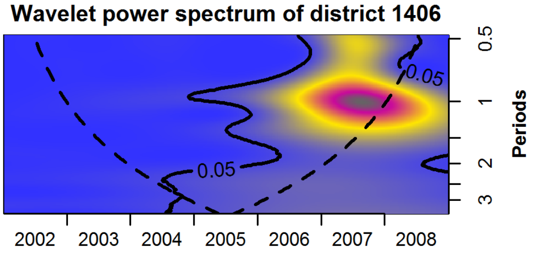

Wavelet power spectrum of district 1407

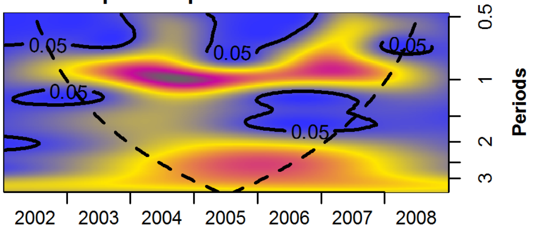

Wavelet power spectrum of district 1408

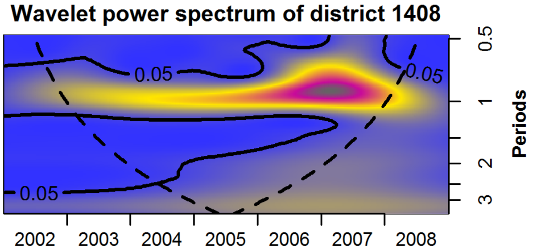

Wavelet power spectrum of district 1409

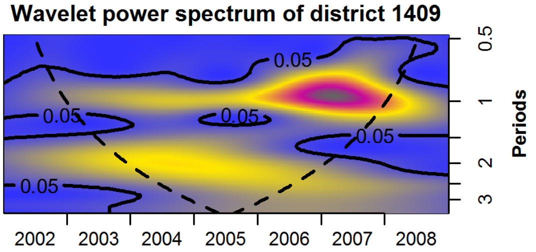

Wavelet power spectrum of district 1410

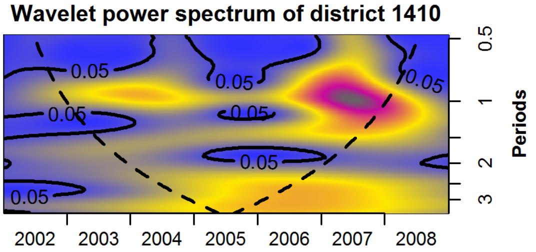

Wavelet power spectrum of district 1411

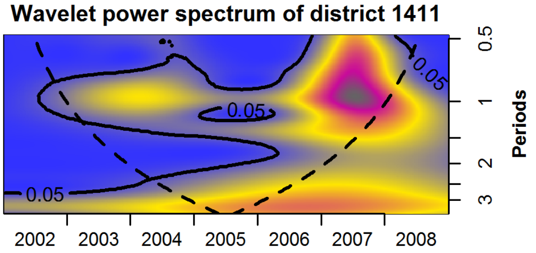

Wavelet power spectrum of district 1412

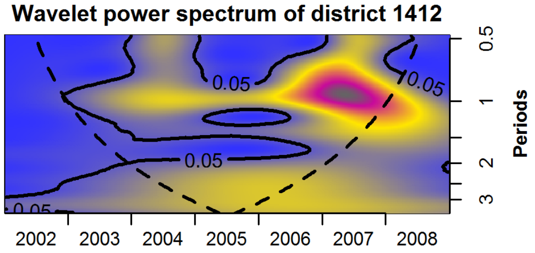

Wavelet power spectrum of district 1501

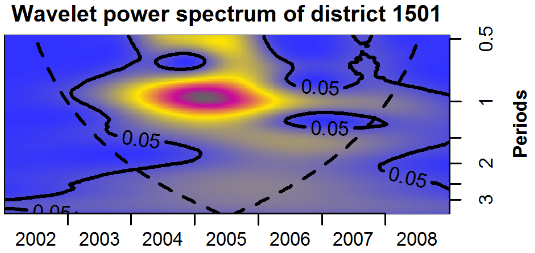

Wavelet power spectrum of district 1502

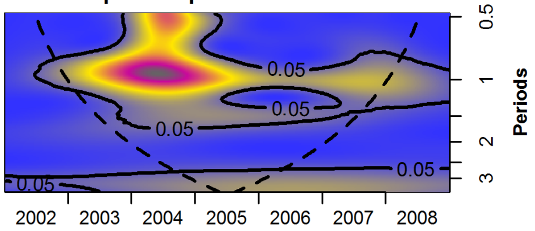

Wavelet power spectrum of district 1503

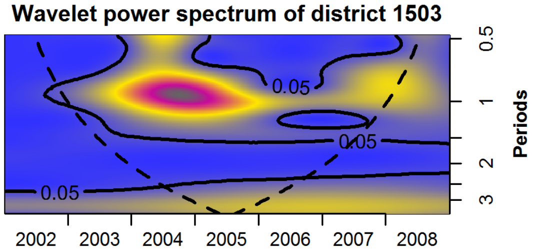

Wavelet power spectrum of district 1505

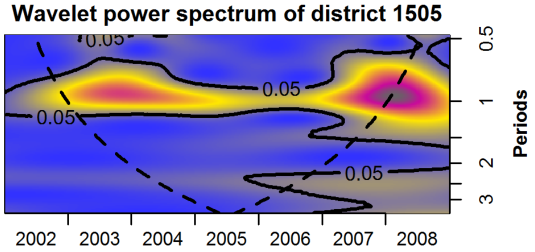

Wavelet power spectrum of district 1701

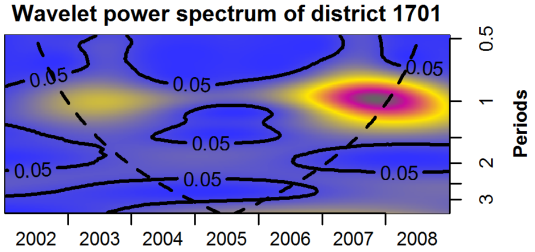

Wavelet power spectrum of district 1702

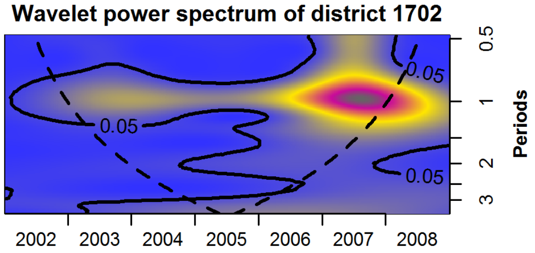

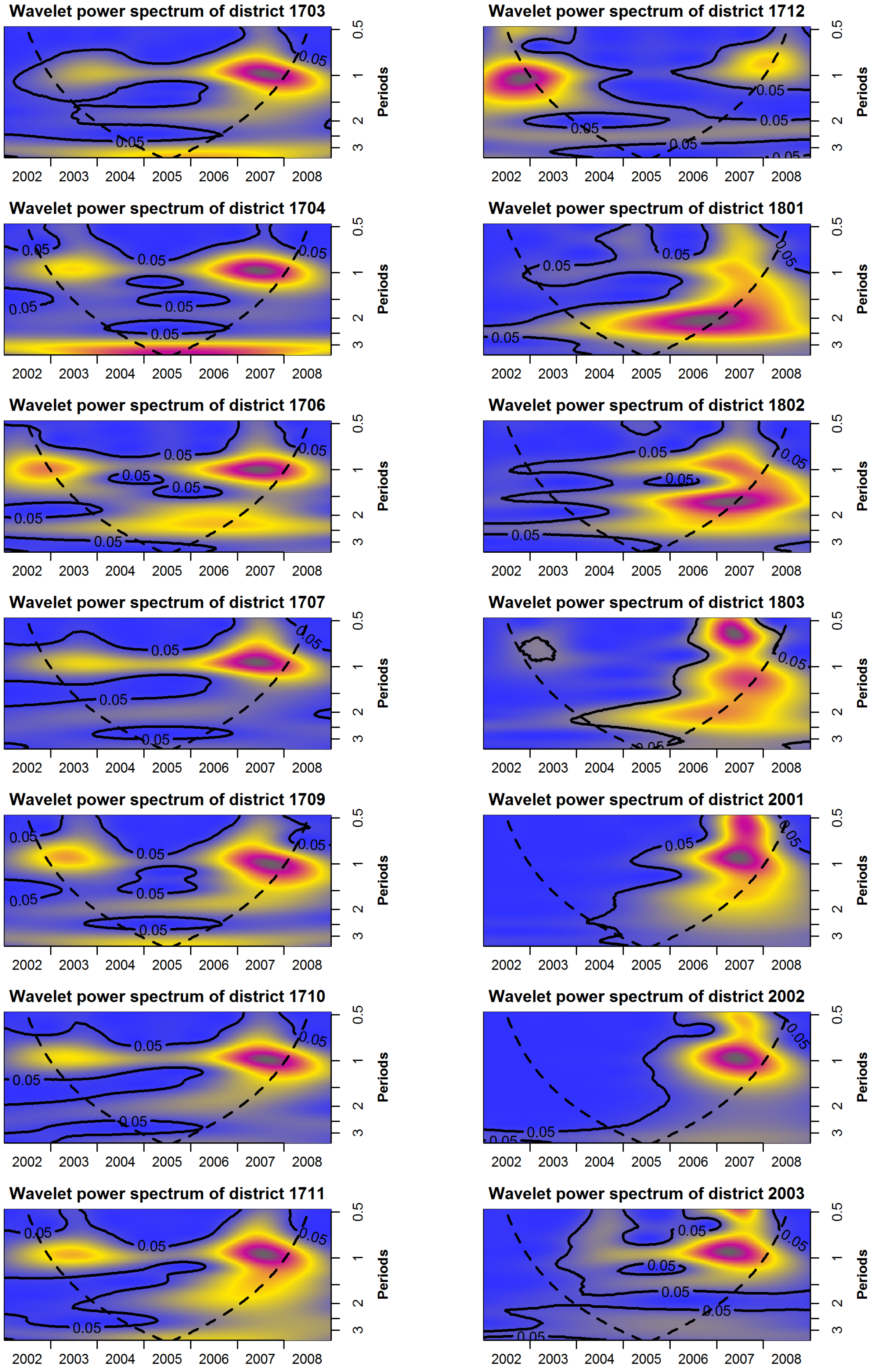
Wavelet power spectrum of district 1703
Wavelet power spectrum of district 1712
Wavelet power spectrum of district 1704
Wavelet power spectrum of district 1801
Wavelet power spectrum of district 1706
Wavelet power spectrum of district 1802
Wavelet power spectrum of district 1707
Wavelet power spectrum of district 1803
Wavelet power spectrum of district 1709
Wavelet power spectrum of district 2001
Wavelet power spectrum of district 1710
Wavelet power spectrum of district 2002
Wavelet power spectrum of district 1711
Wavelet power spectrum of district 2003
2002 2003 2004 2005 2006 2007 2008
Periods
0.5 1 2 3
0.05

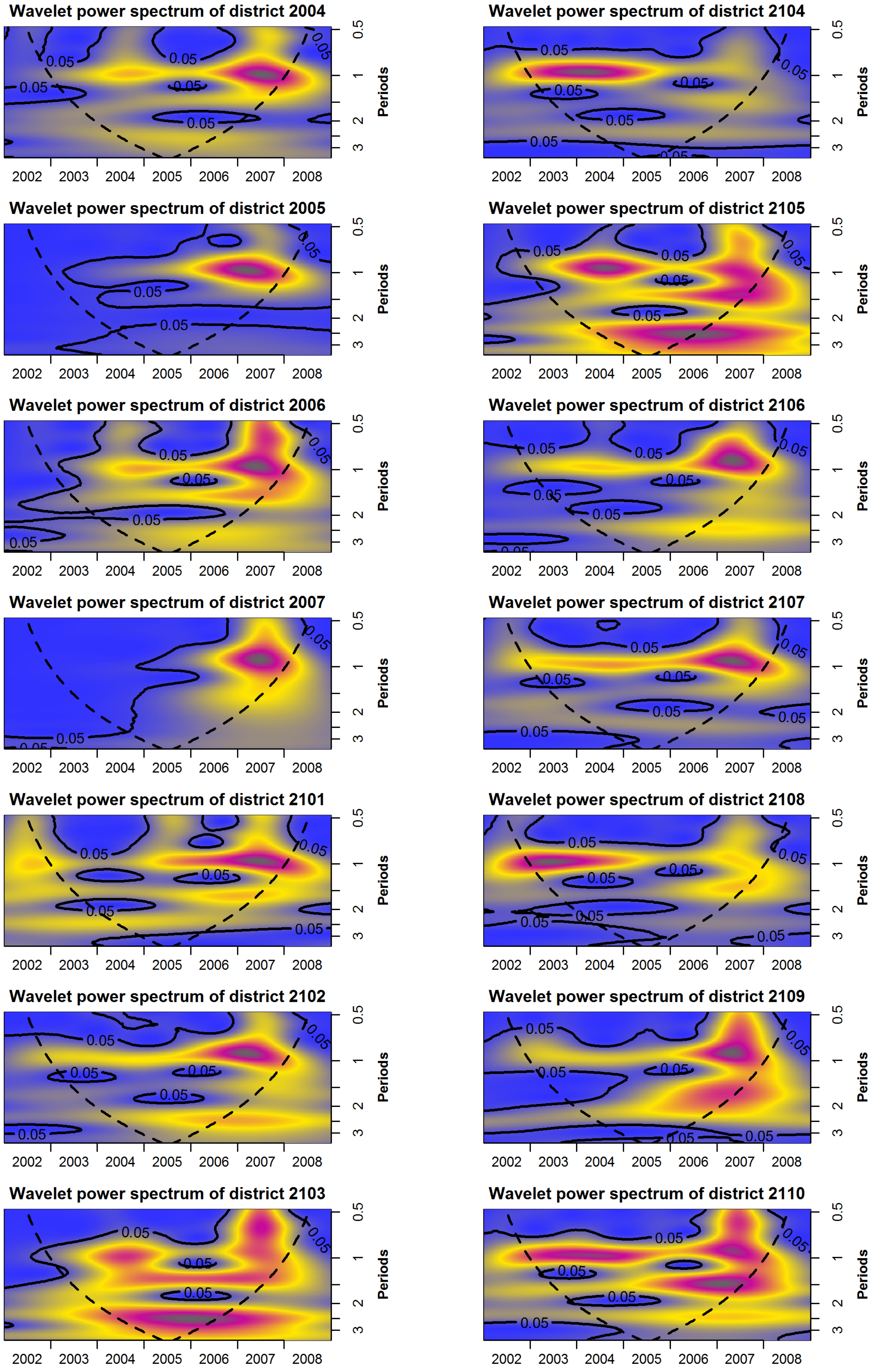
Wavelet power spectrum of district 2004
Wavelet power spectrum of district 2104
Wavelet power spectrum of district 2005
Wavelet power spectrum of district 2105
Wavelet power spectrum of district 2006
Wavelet power spectrum of district 2106
Wavelet power spectrum of district 2007
Wavelet power spectrum of district 2107
Wavelet power spectrum of district 2101
Wavelet power spectrum of district 2108
Wavelet power spectrum of district 2102
Wavelet power spectrum of district 2109
Wavelet power spectrum of district 2103
Wavelet power spectrum of district 2110
2002 2003 2004 2005 2006 2007 2008
0.5 1 2 3
Periods
0.05

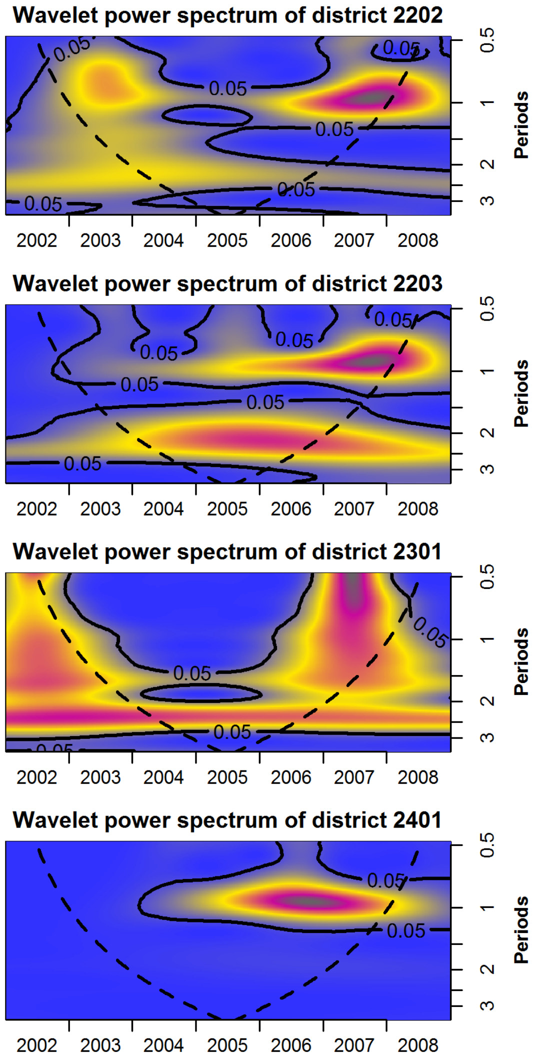
Wavelet power spectrum of district 2202
Wavelet power spectrum of district 2203
Wavelet power spectrum of district 2301
Wavelet power spectrum of district 2401
0.05
Periods
0.5
1
2
3
2002
2003
2004
2005
2006
2007
2008
